# Engineering *Pseudomonas protegens* Pf-5 for Nitrogen Fixation and its Application to Improve Plant Growth under Nitrogen-Deficient Conditions

**DOI:** 10.1371/journal.pone.0063666

**Published:** 2013-05-13

**Authors:** Lorena Setten, Gabriela Soto, Matteo Mozzicafreddo, Ana Romina Fox, Christian Lisi, Massimiliano Cuccioloni, Mauro Angeletti, Elba Pagano, Antonio Díaz-Paleo, Nicolás Daniel Ayub

**Affiliations:** 1 Instituto de Genética Ewald A. Favret (CICVyA-INTA), Castelar, Buenos Aires, Argentina; 2 Consejo Nacional de Investigaciones Científicas y Técnicas (CONICET), Cuidad Autónoma de Buenos Aires, Argentina; 3 School of Biosciences and Biotechnology, University of Camerino, Camerino (MC), Italy; University Paris South, France

## Abstract

Nitrogen is the second most critical factor for crop production after water. In this study, the beneficial rhizobacterium *Pseudomonas protegens* Pf-5 was genetically modified to fix nitrogen using the genes encoding the nitrogenase of *Pseudomonas stutzeri* A1501 via the X940 cosmid. Pf-5 X940 was able to grow in L medium without nitrogen, displayed high nitrogenase activity and released significant quantities of ammonium to the medium. Pf-5 X940 also showed constitutive expression and enzymatic activity of nitrogenase in ammonium medium or in nitrogen-free medium, suggesting a constitutive nitrogen fixation. Similar to *Pseudomonas protegens* Pf-5, *Pseudomonas putida*, *Pseudomonas veronii* and *Pseudomonas taetrolens* but not *Pseudomonas balearica* and *Pseudomonas stutzeri* transformed with cosmid X940 showed constitutive nitrogenase activity and high ammonium production, suggesting that this phenotype depends on the genome context and that this technology to obtain nitrogen-fixing bacteria is not restricted to Pf-5. Interestingly, inoculation of Arabidopsis, alfalfa, tall fescue and maize with Pf-5 X940 increased the ammonium concentration in soil and plant productivity under nitrogen-deficient conditions. In conclusion, these results open the way to the production of effective recombinant inoculants for nitrogen fixation on a wide range of crops.

## Introduction

Nitrogen fertilizer application is an essential input for crop productivity in most regions of the world [1]. Reduction or elimination of this application will attenuate our dependence on fossil fuels as they are necessary for the production of nitrogen fertilizer. Both from health and environmental perspectives, the quality of ground water is adversely affected by nitrogen fertilization [2]. The interest in these problems has prompted the study of biological nitrogen fixation.

Nitrogen fixation is widespread among the Eubacteria and Archae domains [3]. These metabolically divergent and non-phylogenetic groups of microorganisms are collectively known as diazotophs; all of them possess a metallo-enzyme complex, the nitrogenase, which catalyzes one of the most remarkable chemical transformations in biological systems: the ATP-dependent reduction of atmospheric dinitrogen (N_2_) to bioavailable ammonia [4]. In concordance with their exceptionally genetic diversity, diazotrophs have diverse lifestyles and several mechanisms to prevent O_2_ from damaging their nitrogenase. There are numerous free-living strains of bacteria and archaea that are able to fix N_2_ under aerobic (e.g. *Azotobacter vinelandii* and *Anabaena* sp. strain PCC 7120) and anaerobic (e.g. *Klebsiella pneumonia* and *Methanosarcina barkeri*) conditions [5], [6], [7]. In addition, many strains of nitrogen-fixing *Proteobacteria* (e.g. *Ensifer meliloti* and *Cupriavidus taiwanensis), Actinobacteria* (e.g. *Frankia alni* and *Auraticoccus monumenti*) and *Cyanobacteria* (e.g. *Nostoc punctiforme*) can form symbiotic associations with legumes (e.g. soybean and alfalfa) and non-leguminous plants (e.g. shrubs and trees), providing the host with nitrogen-rich compounds [8], [9], [10], [11], [12], [13]. Unfortunately, this type of symbiosis is not observed in the most economically important crops (e.g. maize, rice and wheat) [14].

In this context, finding nitrogen-fixing inoculants to improve the production of important non-leguminous crops has often been touted as one of the ultimate goals of nitrogen fixation research [2], [15]. An approach to mitigate this difficulty is the use of endophytic diazotrophs. For example, Wood and coworkers have shown that 20% of wheat shoots nitrogen has been derived from *Azospirillum brasilense* nitrogen fixation under laboratory co-culture model [16]. Although, robust experiments have shown that endophytic diazotrophs (e.g. *Acetobacter diazotrophicus* and *Pseudomonas stutzeri* A1501) are able to fix nitrogen in the rhizosphere and can efficiently promote the growth of non-leguminous crops (e.g. Sugar cane and Rice), the potential efficiency and the mechanism of transfer of fixed nitrogen from these strains to their plant hosts are still uncertain [2], [14], [16], [17], [18], [19], [20], [21], [22], [23]. In fact, the transfer of fixed nitrogen from plant root-associated diazotrophs to their non-legume hosts has been considered a key problem in the development of more effective nitrogen-fixing inoculants [2].

An approach complementary to the use of natural bacteria as inoculants could be the development of genetically modified organisms (GMOs) derived from plant growth-promoting rhizobacteria (PGPR). For instance, genetic engineering has been applied to increase the production of auxin and antifungal compounds in *Pseudomonas, Azospirillum* and *Bacillus* strains [24], [25], [26], [27], [28], [29], [30], [31]. Regarding genetic engineering for nitrogen fixation, some authors began to investigate the transfer of *nif* genes among different strains belonging to the phylum *Proteobacteria*. Some of these pioneer studies analyzed *nif* transfer between different strains of *Klebsiella pneumonie* via conjugation [32] and transduction [33]. Then, Dixon and Postgate demonstrated for the first time the transfer of a functional nitrogenase between two strains and empirically corroborated the nitrogen fixation in *Escherichia coli* expressing *nif* genes from *Klebsiella pneumoniae* by using the N15 isotope method [34]. Postgate and Kent also demonstrated that *nif* genes from *K. pneumoniae* can be transferred to *Pseudomonas putida* MT20-3 and that the recombinant *Pseudomonas* strain was able to express the *nif* genes and displayed nitrogenase activity quantified by acetylene reduction [35]. This result unambiguously confirmed that *K. pneumoniae nif* genes can be functionally expressed in an obligate aerobic microbe such as strains of the genus *Pseudomonas.* Additionally, it has been described that nodulation and nitrogen fixation genes can be transferred from symbiotic to nonsymbiotic rhizobia via mobile genetic elements [36], [37], [38], [39], [40], [41]. While all these studies have shown the transfer of natural mobile elements containing *nif* genes between different microorganisms, none has helped to develop inoculants.

Several works have shown bioinformatic evidence supporting horizontal transfer of nitrogenase among microorganisms. For example, phylogenetic analyses of *nif* genes have shown incongruence with rRNA data [42], [43], [44] and that *nif* genes are localized within mobile genetic elements, such as the *nif* genes found within the genomic islands, also named nitrogen fixation islands, on the chromosome of *Pseudomonas stutzeri* A1501 and *Pseudomonas stutzeri* DSM4166 [23], [45]. Nitrogen fixation is a rare feature in the genus *Pseudomonas* [46], [47] and the presence of nitrogen fixation islands within some strains belonging to this genus suggests (i) that *Pseudomonas* could be a favorable background for the expression of a heterologous nitrogenase and (ii) that the horizontal transfer of nitrogen fixation islands in *Pseudomonas* strains has occurred recently and that, then, all genes required for the expression of nitrogenase could be efficiently packaged within the nitrogen fixation islands of *P. stutzeri* A1501 and *P. stutzeri* DSM4166. The latter is supported by indirect evidence such as the global transcriptional analysis of nitrogen fixation in *P. stutzeri* A1501 [23], [48].

The aim of the present study was to develop a genetic-engineering technique to obtain recombinant nitrogen-fixing bacteria. In the analysis of *nif* gene transfer performed in this work, the donor (i.e. *Pseudomonas stutzeri* A1501) and recipient (i.e. *Pseudomonas protegens* Pf-5, *Pseudomonas putida* KT2440, *Pseudomonas veronii* DSM11331, *Pseudomonas taetrolens* IAM1653, *Pseudomonas balearica* SP1402 and *Pseudomonas stutzeri* CCUG11256) strains were restricted to well-characterized species belonging to *Pseudomonas sensu stricto* [23], [46], [49], [50], [51], [52], [53], [54], [55], [56]. The key to constructing a recombinant vector containing all the genes necessary for expressing a functional nitrogenase was the use of *P. stutzeri* A1501 as the donor strain because this bacterium has *nif* genes co-localizated within the chromosome [23]. On the other hand, *Pseudomonas protegens* Pf-5 was selected as a recipient strain of *nif* genes because this bacterium is a biological control agent bacterium that lives in the rhizosphere of a wide variety of plant species and is able to persist and compete with native soil microbes [49], [57], [58]. In this paper, we report on the transformation of *P. protegens* Pf-5 with the nitrogen fixation island from *P. stutzeri* A1501 via the recombinant cosmid (X940). The recombinant strain Pf-5 X940 displayed high nitrogenase activity, released significant quantities of ammonium to the medium and promote the growth of *Arabidopsis thaliana*, *Medicago sativa* (alfalfa), *Schenodorus arundinaceus* (tall fescue) and *Zea mays* (maize) under nitrogen deficient-conditions. The characterization of *Pseudomonas putida* KT2440, *Pseudomonas veronii* DSM11331, *Pseudomonas taetrolens* IAM1653, *Pseudomonas balearica* SP1402 and *Pseudomonas stutzeri* CCUG11256 transformed with cosmid X940 suggests that the genetic engineering strategy to obtain nitrogen-fixing strains is not limited to *P. protegens* Pf-5 and can be extrapolated to other bacteria. Hence, this work opens a new perspective for inoculants biotechnology.

## Materials and Methods

### Bacterial Strains

The strains used in this study were *Pseudomonas protegens* Pf-5 [50], [51], *Pseudomonas stutzeri* A1501 [52], *Pseudomonas putida* KT2440 [53], *Pseudomonas veronii* DSM11331 [54], *Pseudomonas taetrolens* IAM1653 [55], *Pseudomonas balearica* SP1402 and *Pseudomonas stutzeri* CCUG11256 [56].

### Recombinant Strain Construction

Two 255-bp fragments from *Pseudomonas stutzeri* A1501 (accession number: CP000304) intergenic regions PST_1306–PST_1307 and PST_1312–PST_1313 were obtained by colony PCR amplification using A1–A2 and A3–A4 primers (Table S1), respectively. The PST_1306–PST_1307 amplification fragment was digested with BamHI, and ligated with kanamycin resistance gene (kan) obtained from plasmid pUC4K (X06404) cut with BamHI. The PST_1312–PST_1313 amplification fragment was digested with SalI and cloned into the recombinant plasmid. The resulting plasmid, which does not replicate in *Pseudomonas,* was introduced by transformation into competent cells of A1501 prepared as previously [59]. Transformants were selected by plating on LB agar containing 50 µg/ml of kanamycin. A single kanamycin colony was chosen and named A1501C. The double recombinant event in strain A1501C was checked by PCR as previously reported [59] and its ability to fix nitrogen was confirmed by growth in L medium without (NH_4_)_2_SO_4_.

The SuperCos1 vector (accession number: M99566.1) was digested with AvaI and religated to eliminate the pSV40-neoR reporter cassette. Next, the resulting vector (named pSC2) was digested with XbaI, dephosphorylated, and digested with BamHI, and then ligated with the DNA fragment obtained from A1501C cut with MboI. The genomic library was screened for the presence of the kanamycin resistance gene, using *Escherichia coli* grown on LB medium containing kanamycin 50 µg/ml and ampicillin 100 µg/ml. One recombinant cosmid, named X940, obtained from the genomic library of A1501C containing *nif* genes was sequenced by primer walking (Dataset S1, Figure S1). The X940 cosmid was introduced by transformation into competent cells of *P. protegens* Pf-5, *Pseudomonas putida* KT2440, *Pseudomonas veronii* DSM11331, *Pseudomonas taetrolens* IAM1653, *Pseudomonas balearica* SP1402 and *Pseudomonas stutzeri* CCUG11256, prepared as previously described [59]. Transformants were selected by plating on LB agar containing 50 µg/ml of kanamycin. The recombinant strains containing the cosmid X940 were named Pf-5 X940, KT2440 X940, DSM11331 X940, IAM1653 X940, SP1402 X940 and CCUG11256 X940.

Plasmid pENZO carrying the PST1307–PST1312 region of *Pseudomonas stutzeri* A1501 was constructed by cloning a 6.23 kb PCR amplification fragment using p07 and p13 primers (Table S1) into pBBR1MCS-3 (accession number: U25059) digested with SmaI. Conjugation of *Pseudomonas protegenes* Pf-5 X940 with *Escherichia coli* S17-1 harboring pENZO plasmid was performed on mineral salts medium plates according to [59]. The resulting recombinant strain derived from Pf-5 and containing cosmid X940 and pENZO plasmid was named Pf-5 X940(2).

To corroborate the presence of the *nif* genes within the recombinant *Pseudomonas* strains, we performed PCR assays (Figure S2). Genomic DNA was isolated from overnight cultures using Wizard Genomic DNA Purification Kit (#A1120, Promega, USA). For the PCR assays, the 950-bp fragments of the *nifH* gene were obtained by PCR amplification using N1 and N2 primers (Table S1) and a program of 34 cycles of 94°C for 1 min, 56°C for 30 s and 72°C for 1 min and a final cycle of 72°C for 10 min.

To study the insertion of X940 cosmid within Pf-5, we performed a genomic walking assay [59] using 2a, 2b, 59a and 59b primers (Table S1). The further study this insertion, a Southern blot assay was carried out. For the last study, a 2-kb digoxigenin-labeled DNA fragment within the PST1302–PST1306 region was generated by PCR by using the PCR DIG probe synthesis kit (Roche) and used as a probe. PCR amplification was performed under standard conditions with a program of 34 cycles of 94°C for 1 min, 50°C for 30 s and 72°C for 2.5 min and a final cycle of 72°C for 10 min. Digoxigenin labeled PCR products were generated for use as probes using oligonucleotides PST1302up and PST1306low (Table S1). Genomic DNA was cut using XhoI or HindIII and DNA fragments in gels were then transferred to a positively charged Nylon membrane (Roche). Nylon membranes were crosslinked and then used for hybridization with DIG-labeled PST1302–PST1306 probe. Prehybridization and hybridization was carried out according to the manufacturer’s instructions (Roche).

### Bacterial Growth under Nitrogen-limiting Conditions

Cultures were performed in 125-ml Erlenmeyer flasks containing 25 ml of L medium, incubated at 28°C with shaking (250 rpm). L medium was prepared as follows: 7.5 mM KH_2_PO_4_, 17.22 mM K_2_HPO_4_, 3.42 mM NaCl, 7.57 mM (NH_4_)_2_SO_4_, 2 mM MgSO_4_ 7 H_2_O, 3.7 mM FeCl_3_ 6 H_2_O, 0.1 mM CuCl_2_ 2 H_2_O, 0.1 mM ZnSO_4_ 7 H_2_O, 0.73 mM MnCl_2_ H_2_O, 1 mM CaCl_2_ 2 H_2_O, 0.21 mM NaMoO_4_, 3.4 mM citric acid, 28 mM glucose, 100 mg/l yeast extract, pH = 7. To test growth under nitrogen-limiting conditions in aerobic (Erlenmeyer covered with parafilm) and micro-aerobic (Erlenmeyer flasks with plastic screw caps) environments, overnight cultures grown in L medium were used to inoculate L medium with or without (NH_4_)_2_SO_4_. All cultures were adjusted to an initial optical density (OD580 nm) of 0.05 (around 10^6^ CFU/ml). The preculture was washed twice in L medium without nitrogen sources and then used to inoculate culture. Bacterial growth was spectrophotometrically monitored at OD580 nm and colony forming units (CFU) counted at 48 h. The identities of the strains were confirmed by PCR colony and subsequently sequenced, using E9F-E1541R, GyrFw-GyrRv and *nif*A-p1 *nif*A-p2 primers for 16S rRNA, DNA gyrase and *nif*A genes, respectively [60], [61], [62].

### Nitrogenase Activity and Ammonium Concentrations in the Medium

Nitrogenase activity was evaluated according to [52] with slight modifications. Bacterial cells from an overnight culture in LB medium containing 50 mM glucose were centrifuged and resuspended in a 50-ml flask containing 10 ml N-free L medium (nitrogen fixation condition) or nitrogen-rich media such as L medium (nitrogen excess conditions) supplemented with 100 mM glucose, at an OD580 nm of 0.1. The suspension was incubated for 8 h at 28°C with shaking under an argon atmosphere containing 1% oxygen and 10% acetylene. The ammonium assay was perfomed using the nitrogen fixation condition described to nitrogenase activity assay but argon was changed for dinitrogen. Ammonium concentrations in the medium were measured by withdrawing a small sample, removing the cells by centrifugation, and assaying the supernatant by the indophenol method described previously [63]. The ethylene production was determined by gas chromatography according to [64] and protein content was determined by the Bradford method [65].

### Inoculation Tests

For the inoculation assay, Columbia-0 *Arabidopsis thaliana*, *Schenodorus arundinaceus* (Festuca Alta Gentos), *Medicago sativa* (alfalfa Forage Genetics 969) and *Zea mays* (maize Pannar Pan6326 RRZ) seeds were surface-sterilized and vernalized for 5 days at 4°C in darkness. Then, plants were incubated in a growth chamber at 23°C under light/dark cycles of 16 h/8 h, with light intensities of 150 µmol m^−2^ sec^−1^, in cultures containing a mixture of peat, perlite and vermiculite (1∶1:1 v/v), a mixture of perlite and vermiculite (1∶1 v/v), 100% vermiculite or a mixture of perlite and soil (1∶4 v/v), respectively. Arabidopsis, tall fescue and alfalfa plants were grown in INTA13 medium (0.88 mM CaCl_2_ 2 H_2_O, 1 mM MgSO_4_ 7 H_2_O, 1 mM Na_2_HPO_4_, 0.73 mM KH_2_PO_4_, 4.13 µM Na_2_MoO_4_ 2 H_2_O, 3.55 µM MnSO_4_ H_2_O, 4 µM CuSO_4_ 5 H_2_O, 3.48 µM ZnSO_4_ 7 H_2_O, 16.17 µM H_3_BO_3_, 14.8 µM FeCl_3_ 6 H_2_O, pH = 6.5) with or without 1 mM Ca(NO_3_)_2_ 4 H_2_O as nitrogen source for 40 days. Each week a 0.5 ml sample of hydroponic medium was taken and the ammonium was measured as described above. The trays and pots were sterilized with 70% ethanol. The substrate was sterilized by heat treatment (350°C for 30 minutes) and washed twice with sterile distilled water. Maize plants were irrigated with water for 30 days. To assay maize growth under nitrogen excess conditions, soil was supplemented with 400 mg NH_4_SO_4_. Arabidopsis, tall fescue and alfalfa plants were inoculated after vernalization either with wild-type (Pf-5) or recombinant bacteria (Pf-5 X940) prepared as follows: 1 ml of overnight cultures grown at 28°C in L medium was centrifuged and then resuspended in 1 ml of saline solution. Then, 200 µl of the bacterial solution (8 10^8^–2 10^9^ colony forming units (CFU)/ml) was used to inoculate plants in pots (1L). We recovered the strains after the inoculation trials. We also detected no contamination in hydroponic medium assays. The identities of the strains were established by PCR colony and subsequently sequenced using E9F-E1541R primers for 16S rRNA gene [62]. In the case of maize inoculation, 2 ml of the bacterial solution was used to inoculate pots of 10 L. Values represent mean+SEM, *n* = 3 independent experiments. Each experiment had 12 plants. Thus, we evaluated 36 plants for each treatment. Soil mineral-N determinations were performed in Laboratorios Fox (http://www.foxlab.com.ar/). NH_4_^+^ was extracted with 1 M KCl (soil:extractant ratio 1∶4) and NO_3_^−^ with water (soil: extractant ratio 1∶2.5) at 250 rpm for 1 h. After centrifuging (4000 rpm for 30 min), supernatants were filtered and mineral-N was determined. NH_4_^+^-N and NO_3_^–^N were quantitatively determined by colorimetric methods (AQAassay, GTLab, Argentina) based on two different sodium salicylate reactions [66], [67]. Absorbance was measured in a DR6000 spectrophotometer (Hach). We evaluated the persistence of Pf-5 and Pf-5 X940 in sterile (121°C, 1 atm, 20 min) soil microcosms (50 g of dry soil) prepared as previously described [68].

### RT-PCR Analysis

Total RNA was extracted by using the RNeasy Mini kit (Qiagen) and treated with DNaseI. cDNA was obtained using random hexamers (Promega) and AMV Reverse Transcriptase M9004 (Promega) following the manufacturer’s instructions. For PCR amplification, 1 µl of RT reaction was used. The PCR reactions were carried out in 25 µl with 0.5 µM of each primer [69]. RT-PCR experiments were performed according to [70] with very slight modifications. PCR was performed by using 1 U Taq Platinum ADN polymerase (Invitrogen), buffer Taq Platinum 10X, 0.25 mM dNTP, 0.4 mM primers and 2 mM MgCl_2_ with Opticon2 (MJ Research), according to the manufacturer’s instruction. Table S1 showed primers used for PCR. PCR conditions comprised: 1 cycle at 94°C for 3 min, 34 cycles of 94°C for 45 s, 59.1°C for 1 min and 72°C for 1 min. The expression of *nifA*, *nifB* and *nifH* genes was normalized to the expression of housekeeping gene gap-1 [71]. This analysis was performed three hours after incubation in L medium. The efficiency of primer binding was determined by linear regression by plotting the cycle threshold (C_T_) value versus the log of the cDNA dilution [72]. qPCR experiments were independently performed three times, with comparable results. The three PCR reactions were carried -out in duplicate.

### Homology Modeling of *Pseudomonas stutzeri* A1501 Nitrogenase

Based on the amino-acid sequence of *Pseudomonas stutzeri* A1501 nitrogenase, the prediction of its unknown three-dimensional structure, chains A and B, was performed using Swiss-Model homology modeling server [73], as previously reported by Mozzicafreddo [74]. In particular, we used Swiss-Pdb Viewer (version 4.01) to create the project files submitted to the server with default parameters settings (BLAST search P value <0.00001 and global degree of sequence identity SIM >25%). Moreover, 1m1nA and 1m1nB from *Azotobacter vinelandii* [75], sharing 91% sequence identity with respect to *P. stutzeri,* were selected as structure templates, producing a high-accuracy comparative model (sequence identity >50%). Nitrogenase query sequences (A4VJ71 for chain A and A4VJ72 for chain B) were obtained from UniProt Knowledgebase (http://beta.uniprot.org/).

## Results

### Introduction of *nif* Genes into *Pseudomonas protegens* Pf-5

**Figure 1:**
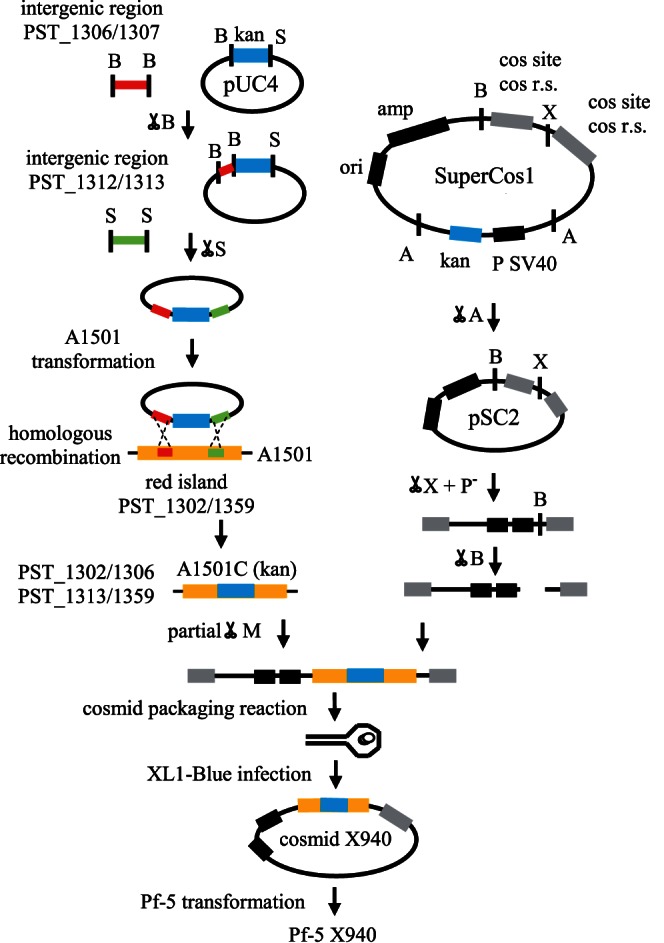
General scheme of the construction of cosmid X940. *Pseudomonas protegens* Pf-5 (Pf-5), *Pseudomonas stutzeri* A1501 (A1501), kanamycin resistance gene (kan), ampicillin resistance gene (amp), BamHI (B), SalI (S), XbaI (X), MboI (M), AvaI (A), dephosphorylation (P**^-^**) are shown.

**Figure 2:**
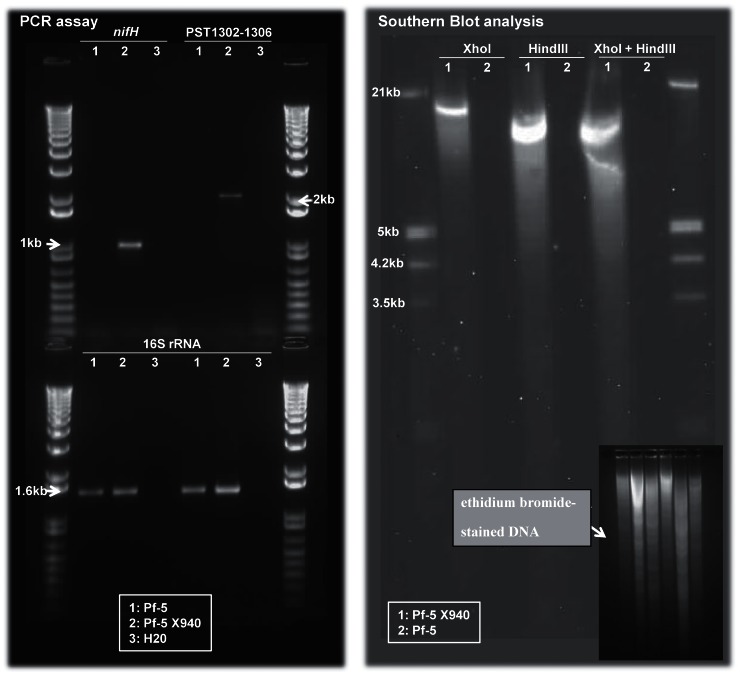
PCR and Southern blot analyses of genomic DNA from the Pf-5 X940 strain carrying the *nifH* gene and the PST1302–PST1306 region. PCR marker: 1 Kb Plus DNA Ladder (INVITROGEN). Southern blot marker: Dig marker III (Roche).

Transfer of the *nif* genes into *Pseudomonas protegens* Pf-5 required several steps of genetic engineering (Figure 1). First, we performed a mutagenesis directed over *Pseudomonas stutzeri* A1501, where the genomic region PST1307–PST1312 was replaced by a kanamycin cassette, resulting in the recombinant strain A1501C. Then, we constructed a recombinant cosmid (pSC2) from SuperCos1 cosmid. pSC2 was identical to the original one, but suffered a deletion in the region that encodes the gene for kanamycin resistance. pSC2 was used to produce a genomic library of the A1501C. After a selection on kanamycin (*nif* region) and ampicillin (vector), we selected X940 cosmid for having 52 genes corresponding to the PST1302–PST1306 and PST1313–PST1359 regions. Cosmid X940, which does not replicate in *Pseudomonas,* was introduced by transformation into *P. protegens* Pf-5. The recombinant *P. protegens* Pf-5 containing cosmid X940 was named Pf-5 X940. Genomic PCR analysis confirmed the presence of the *nifH* gene in the Pf-5 X940 strain and the Southern blot assay showed that the recombinant strain has only one copy of cosmid X940 inserted within its genome (Figure 2). Genome walking assays demonstrated that this insertion is within the PFL_0092– PFL_0093 intergenic region (Figure S3).

### Growth of Pf-5 X940 under Nitrogen Limited Conditions

**Figure 3:**
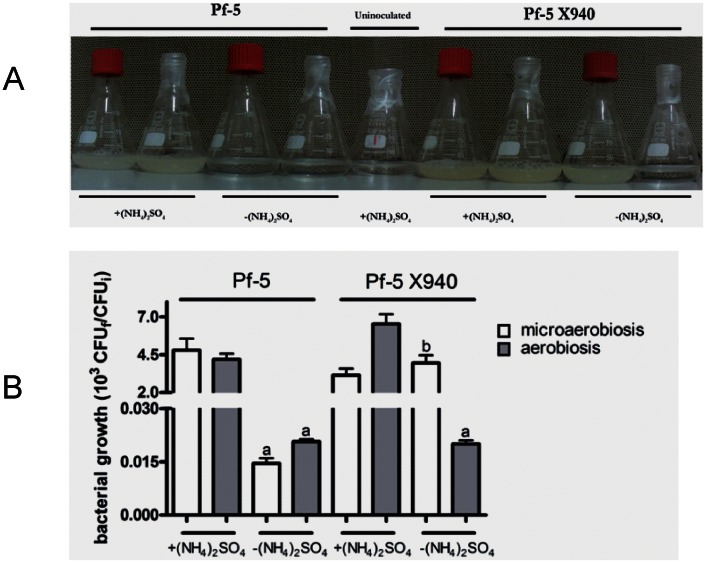
Effect of the transformation of *Pseudomonas* Pf-5 with cosmid X940. We analyzed the growth of Pf-5 and Pf-5 X940 in L medium with and without nitrogen under microaerobiotic (Erlenmeyers with lid) and aerobic (Erlenmayers sealed with parafilm) conditions by means of turbidity (a) and the relationship between the CFU (colony-forming units) at the beginning and at the end of the experiment (b). The statistical analysis was carried out with ANOVA followed by Tukey’s contrast test. The letters correspond to the treatments compared. The same letters correspond to non-significant differences. (a) and (b) showed significant differences with p<0.001.

To study the functionality of the heterologous nitrogenase complex, the growth of both wild-type (Pf-5) and recombinant (Pf-5 X940) bacteria was evaluated by turbidity of the culture medium (Figure 3a) and by counting colony forming units (CFU) (Figure 3b), for 48 hours in L medium either without nitrogen (−(NH_4_)_2_SO_4_) or supplemented with nitrogen (+(NH_4_)_2_SO_4_), in aerobiosis or microaerobiosis under laboratory conditions. During the first 24 hours, growth was observed only in the cultures supplemented with nitrogen (data not shown), whereas after 48 hours significant growth was also observed in the Pf-5 X940 bacterium in L medium without nitrogen under microaerobic conditions (Figure 3). This recombinant bacterium showed no significant growth in L medium without nitrogen in aerobic conditions (Table S2), suggesting that the heterologous nitrogenase complex was active only at low oxygen tension (Figure 3). Under microaerobiosis, the number of Pf-5 bacteria increased two-fold (probably due to a residual cell division), whereas the number of Pf-5 X940 bacteria increased more than three orders of magnitude (approximately 12 generations) in L medium without nitrogen (Figure 3b), suggesting that the recombinant strain could use molecular nitrogen (N_2_) as nitrogen source.

### Nitrogenase Activity and *nif* Genes Expression of Pf-5 X940

**Figure 4:**
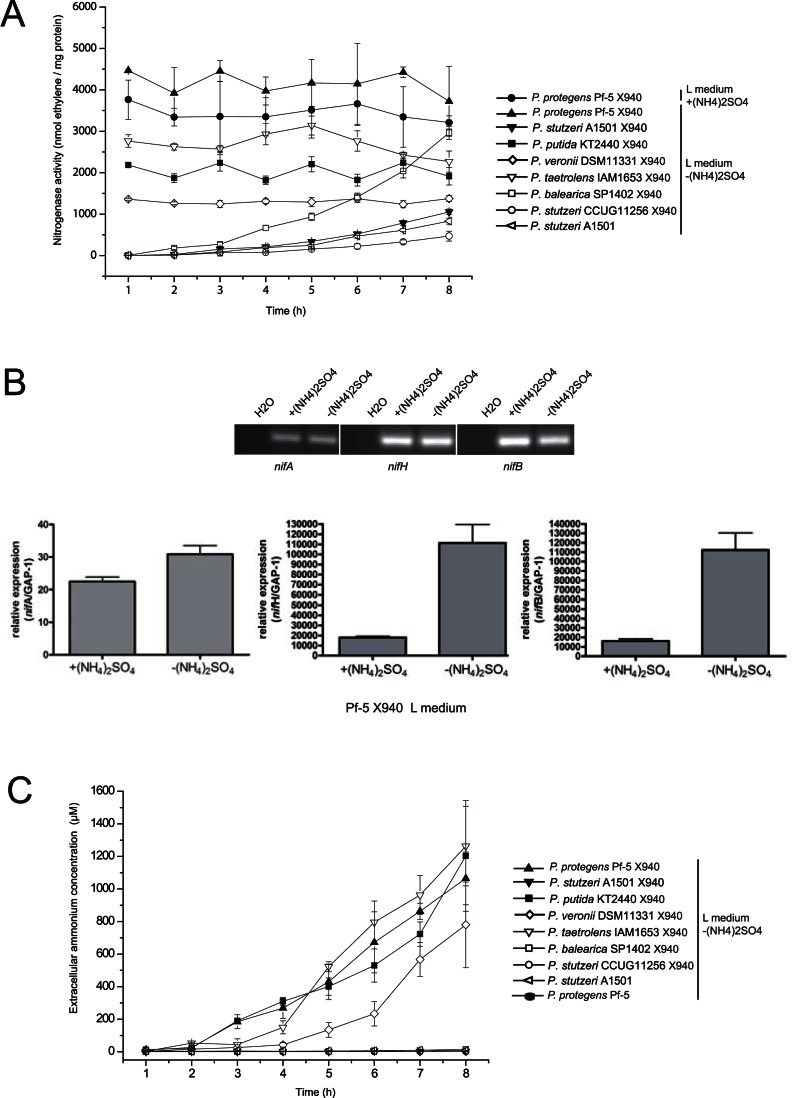
Adaptation of nitrogenase to new bacteria (a) nitrogenase activity, (b) *nif* gene expression and (c) ammonium production of recombinant *Pseudomonas* containing X940 cosmid. Nitrogenase activity and extracellular ammonium concentration values are the mean±SEM of three independent measurements. RT-PCR and Real-time RT-PCR (*nifA*, *nifH* and *nifB*) studies of *nif* gene expression in Pf-5 X940 in the presence (+(NH_4_)_2_SO_4_) or absence (−(NH_4_)_2_SO_4_) of nitrogen. Transcript abundance for *nifA*, *nifH* and *nifB* genes was normalized to that for GAP-1**gene. Values represent media+SEM, n = 3 independent experiments.

To characterize the heterologous nitrogenase complex, we analysed nitrogenase activity in Pf-5 X940 (PST1302–PST1306 and PST1313–PST1359) in L medium in the presence or absence of nitrogen under microaerobiosis. As expected, no nitrogenase activity was observed in wild-type bacteria Pf-5 (<1 nmol ethylene/h/mg protein). In contrast, the recombinant bacteria containing *nif* genes showed nitrogenase activity (Figure 4a). More importantly, Pf-5 X940 displayed an uncommon nitrogenase activity phenotype. First, nitrogenase activity was not significantly repressed in this recombinant strain in medium containing ammonium (Figure 4a). Second, Pf-5 X940 did not show a typical desrepression phenotype observed in natural nitrogen-fixing strains such as A1501 in L medium in the absence of ammonium but displayed a constitutive nitrogenase activity (Figure 4a). In concordance with this unusual phenotype, we could detect the expression of genes coding for positive transcriptional regulator (*nifA*), structural (*nifH*) and biosynthetic (*nifB*) components of the nitrogenase complex in L medium supplemented with ammonium (Figure 4b), suggesting that Pf-5 context was unable to completely repress the transcription of *nif* genes under nitrogen excess conditions. However, the addition of ammonium had a negative effect on the expression of *nif* genes in Pf-5 X940 (Figure 4b), suggesting that this recombinant strain could retain at least some of the negative transcriptional regulation under nitrogen excess conditions. In addition, constitutive nitrogenase activity in the recombinant strain was associated with high ammonium production in the L medium without nitrogen (Figure 4c). Moreover, the recombinant strain Pf-5 X940(2) containing the PST1302–PST1359 *nif* region displayed a nitrogenase activity, an ammonium production and a *nif* gene expression pattern similar to Pf-5 X940 (Figure S4), suggesting that the PST1307–PST1312 region is not involved in adaptation of *nif* genes to the new host.

To assess whether it is possible to transfer to other strains the ability to fix nitrogen through the genetic engineering methodology developed in this work, we transformed the strains *Pseudomonas putida* KT2440, *Pseudomonas veronii* DSM11331, *Pseudomonas taetrolens* IAM1653, *Pseudomonas balearica* SP1402 and *Pseudomonas stutzeri* CCUG11256 with cosmid X940 (Figure S3). All strains showed nitrogenase activity (Figure 4a), suggesting that this methodology may have applications not restricted to the Pf-5 strain. More interestingly, we observed that some strains, such as *P. balearica* SP1402 X940 and *P. stutzeri* CCUG11256 X940, showed a classic desrepresion-pattern of nitrogenase activity as seen also in the control strain A1501, but other strains of this genus, such as *P. putida* KT2440 X940, *P. veronii* DSM11331 X940 and *P. taetrolens* IAM1653 X940 showed a nitrogenase activity pattern similar to that observed in Pf-5 X940 (Figure 4a). In association with constitutive nitrogenase activity, *P. putida* KT2440 X940, *P. veronii* DSM11331X940 and *P. taetrolens* IAM1653 X940 showed high ammonium production in the L medium without nitrogen (Figure 4c). These results strongly suggest that the adaptation of the nitrogenase complex depends on the genome context.

### Effect of Inoculation with Pf-5 X940 on Plant Productivity

**Figure 5:**
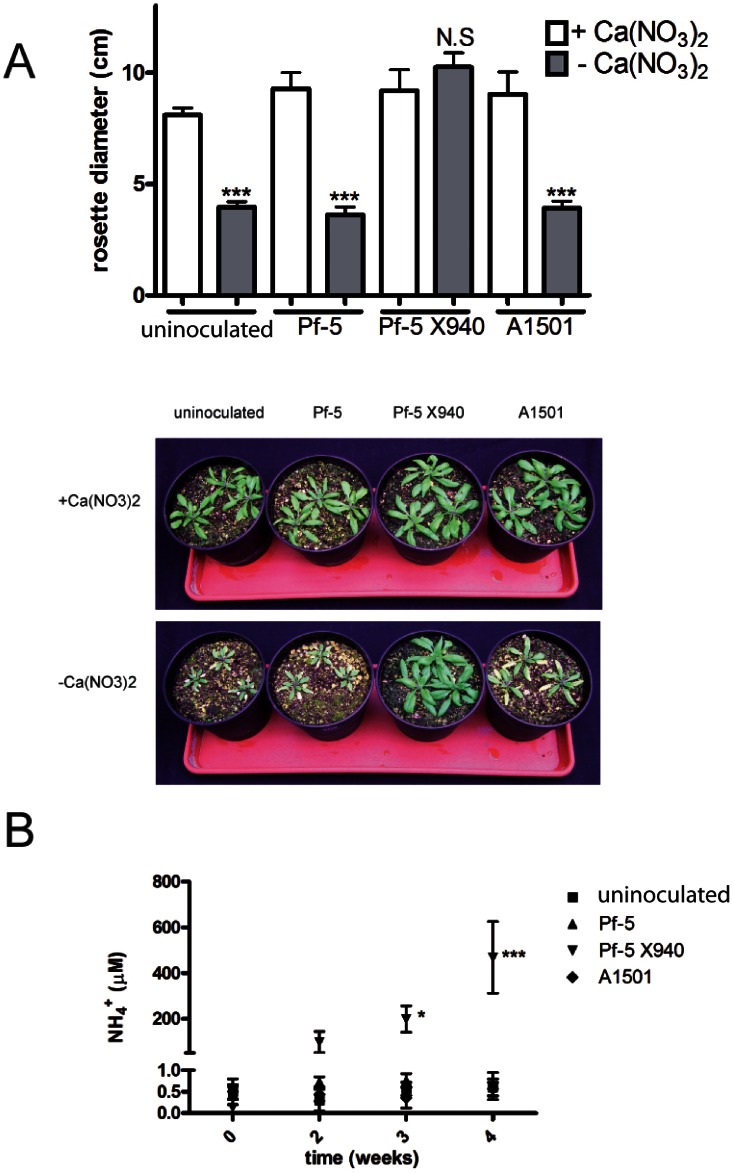
Effect of the inoculation with Pf-5 X940 on (a) Arabidopsis growth and (b) ammonium production. We analyzed the productivity (rosette diameter) and measured the ammonium in solid (NH_4_^+^) of Arabidopsis plants inoculated with A1501, Pf-5 and Pf-5 X940 in the presence (+Ca(NO_3_)_2_) or absence (−Ca(NO_3_)_2_) of nitrogen in the substrate. The statistical analysis was carried out with two-way ANOVA in the case of ammonium measurements or one-way ANOVA in the case of rosette diameter, followed by Tukey’s multiple comparison tests in both cases. Comparisons were made between plants that received the same inoculation treatment in different conditions of nitrogen in the substrate (***p<0.001 **p<0.01 and *p<0.05). N.S: not significant.

To analyze the potential impact of the Pf-5 X940, we assessed the effect of the inoculation with Pf-5 X940 on the growth of *Arabidopsis* (Figure 5). This plant was grown in hydroponics and irrigated with the minimal medium called “INTA13” either without nitrogen (−Ca(NO_3_)_2_) or supplemented with nitrogen (+Ca(NO_3_)_2_), inoculated with Pf-5, Pf-5 X940, A1501, or non-inoculated (control).

**Figure 6:**
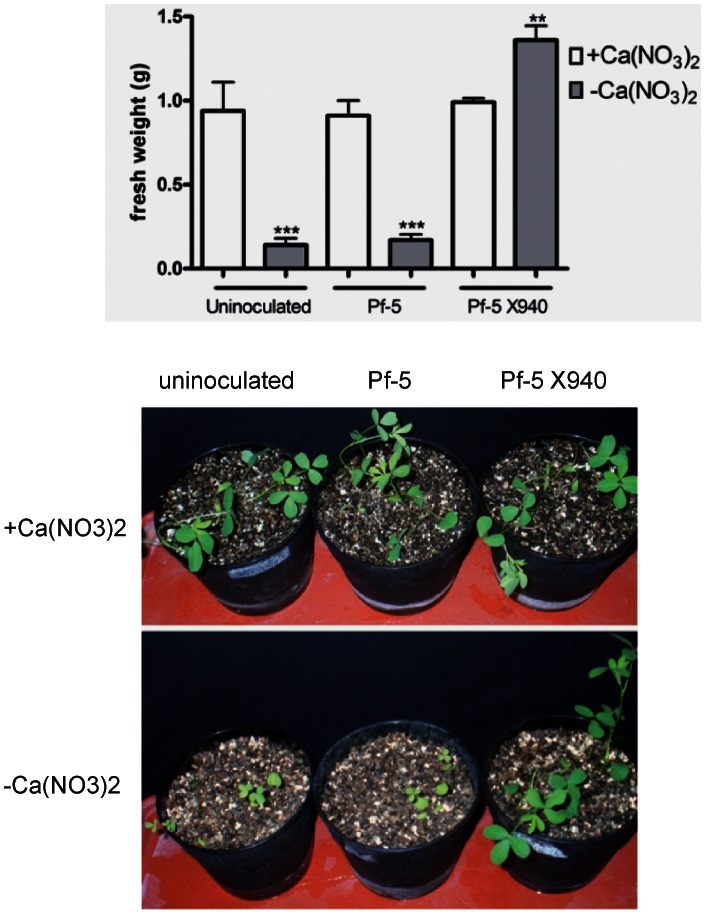
Effect of the inoculation with PF-5 X940 on alfalfa growth. We analyzed the productivity (fresh weight) of plants inoculated with Pf-5 and Pf-5 X940 in the presence (+Ca(NO_3_)_2_) or absence (−Ca(NO_3_)_2_) of nitrogen in the substrate. The statistical analysis was carried out with ANOVA followed by Tukey’s contrast test. Comparisons were made between plants that received the same inoculation treatment in different conditions of nitrogen in the substrate (**p<0.01 and ***p<0.001).

**Figure 7:**
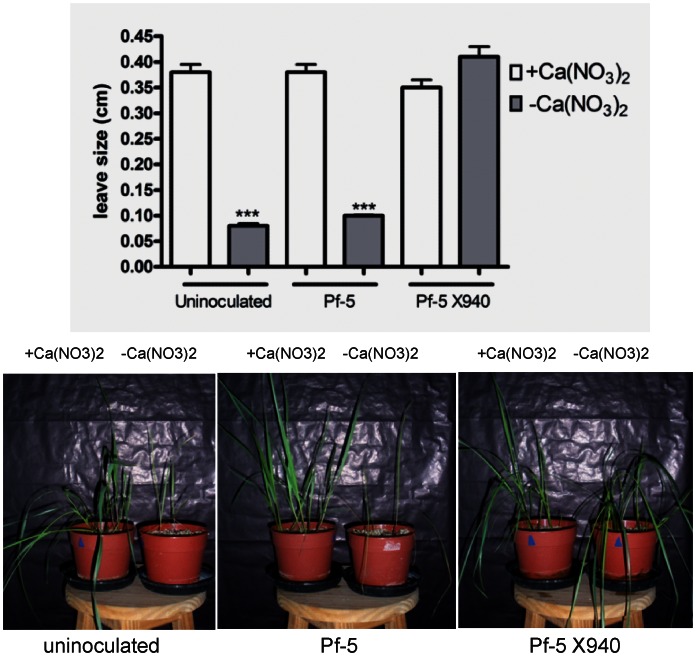
Effect of the inoculation with PF-5 X940 on tall fescue growth. We analyzed the productivity (first leaf width) of plants inoculated with Pf-5 and Pf-5 X940 in the presence (+Ca(NO_3_)_2_) or absence (−Ca(NO_3_)_2_) of nitrogen in the substrate. The statistical analysis was carried out with ANOVA followed by Tukey’s contrast test. Comparisons were made between plants that received the same inoculation treatment in different conditions of nitrogen in the substrate (**p<0.01 and *p<0.05).

After 40 days of growth, the non-inoculated plants and plants inoculated with the wild-type strains showed a significantly lower productivity in INTA13 medium without nitrogen than in this same medium supplemented with nitrogen (Figure 5A). This growth reduction in the medium without nitrogen was completely reversed by inoculation with the recombinant bacterium Pf-5 X940 (Figure 5A). This reversion was positively associated with the ammonium levels (Figure 5B), suggesting that the beneficial effect of Pf-5 X940 inoculation could be attributed to the production and excretion of a high amount of ammonium to the medium. No significant differences in plant growth were observed with three different inoculants when the medium was supplemented with nitrogen (Figure 5A). Similar positive effects of Pf-5 X940 inoculation were observed in two agronomicaly important plants, the dicotyledenous *Medicago sativa* (alfalfa) (Figure 6) and the monocotyledoneous *Schenodorus arundinaceus* (tall fescue) (Figure 7) under hydroponic conditions. Unexpectedly, the productivity of alfalfa inoculated with Pf-5 X940 and grown in INTA13 medium without nitrogen was even significantly greater than that found in non-inoculated plants and plants inoculated with Pf-5 and grown in INTA13 with nitrogen (Figure 6).

**Figure 8:**
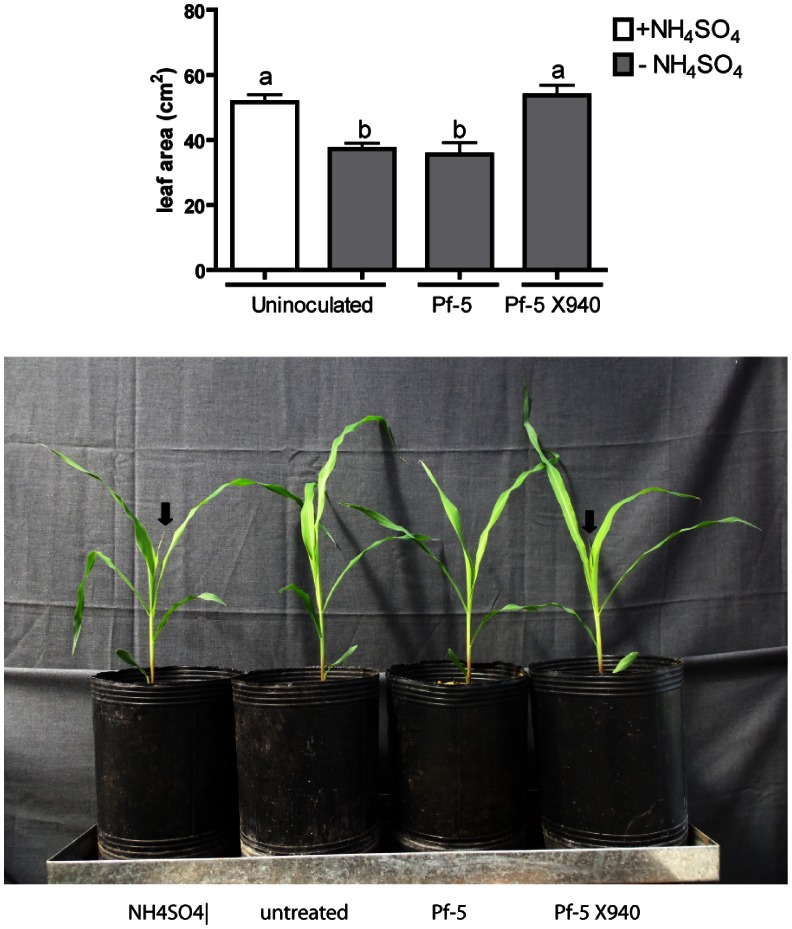
Effect of the inoculation with PF-5 X940 on maize growth. We analyzed the productivity (the area of the third leaf) of plants inoculated with Pf-5 and Pf-5 X940 in the presence (+NH_4_SO_4_) or absence (−NH_4_SO_4_) of nitrogen in the soil. Arrows indicate the presence of the fourth leaf. The statistical analysis was carried out with ANOVA followed by Tukey’s contrast test (***p<0.001).

**Figure 9:**
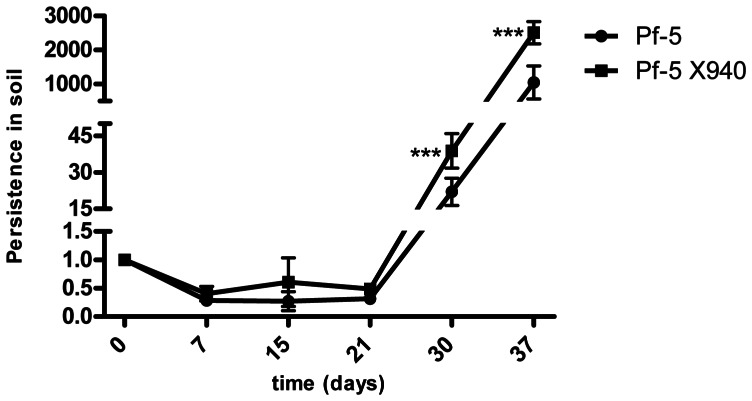
Persistence of bacteria in soil. Sterile soil was inoculated with Pf-5 or Pf-5 X940. Persistence was expressed as a percentage of the number of colony forming units at time zero, that was taken as 1. The experiment was repeated three independent times. The statistical analysis was carried out with t-test (***p<0.001).

The effect of Pf-5 X940 inoculation was also analyzed in one of the most important crops in the world: *Zea mays* (maize). Pf-5 X940 was able to increase maize productivity after 30 days of growth in soil conditions (Figure 8). Importantly, this phenotype was also associated with an increase in nitrogen compounds (ammonium and nitrate) in soil (Table 1). In addition, Pf-5 and Pf-5 X940 showed no significant differences of persistence under soil microcosms (Figure 9). These results suggest that inoculation with Pf-5 X940 could be an effective strategy to increase the productivity of crops in nitrogen-poor soils.

**Table 1:** Quantification of ammonium and nitrate concentration in maize cultivation soil treated or not (uninoculated) with nitrogen (ammonium), wild-type strain (Pf-5) and recombinant bacterium (Pf-5 X940).

Treatment	Nitrogen compounds in maize cultivation soil (µM)	
	Ammonium	Nitrate
Ammonium	2.77±0.02	4.77±0.81
Uninoculated	2.45±0.03	1.44±0.11
Pf-5	2.59±0.08	0.57±0.81
P-5 X940	295±490	110±100

All values are the mean±SD of two independent measurements.

## Discussion

The genus *Pseudomonas* has gone through many taxonomic modifications over the past 50 years. Thus, many bacteria were moved to other genera, families and orders [46], [76], [77], [78], [79], [80]. Earlier studies considered that there were not any nitrogen-fixing strains within the genus *Pseudomonas*. In fact, the inability to fix nitrogen by *Pseudomonas* species had been proposed as an important taxonomic character [46], [47]. However, recent studies have demonstrated that some strains belonging to the genus *Pseudomonas sensu stricto*, such as *Azotobacter vinelandii* AvOP, *Pseudomonas stutzeri* A1501, *Pseudomonas stutzeri* DSM4166, *Pseudomonas azotifigens* 6HT33b^T^ and *Pseudomonas* sp. K1, do have the capability to fix nitrogen [23], [45], [81], [82], [83], [84], [85]. In addition, the genetic organization of the *nif* genes in *P. stutzeri* shows a high degree of similarity to that of *A. vinelandii* AvOP [23], and the homology-modeled three-dimensional structure of the *P. stutzeri* nitrogenase, based on the published x-ray crystal structure of *A. vinelandii* nitrogenase, confirmed that the catalytic sites of these nitrogenases are extremely conserved (Figure S5). Furthermore, in both *Pseudomonas stutzeri* strains, the *nif* genes have been found within genomic islands [23], [45]. The localization within these mobile elements suggests that these genes were acquired by horizontal transfer via genomic islands.

Despite the advances in genetic engineering previously revealed [32], [33], [34], [35], [36], [37], [38], [39], [40], [41], [46], genetic modification approaches for the development of nitrogen-fixing inoculants derived from strains unable to fix nitrogen have not yet been explored. The main obstacle to meet the controlled and efficient transfer of *nif* genes between strains may be that the minimum number of essential genes needed for the biosynthesis of a functional nitrogenase are not yet known exactly. Even without considering this constraint, the biosynthesis of nitrogenase requires a large set of genes (at least 16 *nif* genes) and this vast set of *nif* genes are usually localized in different regions of the bacterial genome [3], [86], [87]. Thus, the construction of a transferable and easily manipulated vector (such as plasmids and cosmids) containing all *nif* genes required to produce an active nitrogenase is a genetic engineering challenge.

In the present work, we used *Pseudomonas protegens* Pf-5 as the receptor of nitrogen-fixation genes from *Pseudomonas stutzeri* A1501 (Figure 1, Figure 2). As the complete genome of Pf-5 is available [49], [88], we can state that this strain has no nitrogen-fixation genes. Therefore, the growth in the absence of nitrogen, the expression of *nif* genes and the nitrogenase activity observed in the recombinant bacterium Pf-5 X940 is strong empirical evidence of the transfer of a functional nitrogenase complex between two *Pseudomonas* strains (Figure 3, Figure 4). This controlled and efficient transfer system of *nif* genes will allow for assessment of the minimum number of essential genes required to form a functional nitrogenase complex. This can be achieved through deletions of different regions of the cosmid X940 or through new constructions. This new model of transfer will also allow studying how nitrogen-fixation genes adapt to different hosts, from gene transcription to the final assembly of the nitrogenase complex [89], [90], [91]. In addition, the results described in the present work support the horizontal transfer hypothesis previously suggested by phylogenetic analyses.

In *P. stutzeri* A1501 and other diazotrophic *Proteobacteria*, the expression of *nif* genes is positively controlled by a specific alternative sigma factor named NifA. The *nifA* gene (PST1313) of *P. stutzeri* A1501 is present within a nitrogen fixation island (PST1306–PST1359). On the other hand, the mechanism for post-transcriptional modification of nitrogenase in *P. stutzeri* A1501 has not yet been described, but several studies have suggested a nitrogenase “switch off” and “switch on” after ammonium shock and nitrogen deficiency in this strain, respectively [52], [57]. Here, we observed constitutive expression of *nif* genes and unaltered activity of nitrogenase in the recombinant strain *P. protegens* Pf-5 X940 in the presence or absence of nitrogen in the medium (Figure 4). More specifically, we showed expression of the *nifA* gene in Pf-5 X940 growing on medium supplemented or not with ammonium (Figure 4). Considering this result, the expression pattern of *nif* structural (*nifH*) and biosynthetic (*nifB*) genes in this recombinant strain is not unexpected (Figure 4). In this context, it could be attractive to explore the role of native alternative sigma factors in the positive control of the expression of *nif* genes directly and/or via NifA. Here, we also showed that nitrogenase regulation depends on the genome context (Figure 4). Although our study does not describe the mechanisms underlying ammonium-repressed nitrogenase in *P. stutzeri* A1501, it is possible that the recombinant *Pseudomonas* strains developed in this study and the new strains derived from the latter will help to further explore this issue.

In association with the constitutive nitrogenase activity, *P. protegens* Pf-5, *P. putida* KT2440, *P. veronii* DSM11331 and *P. taetrolens* IAM1653 but not *P. balearica* SP1402 and *P. stutzeri* CCUG11256 transformed with X940 release large amount of ammonium to the medium (Figure 4), suggesting that the manipulation of *nif* expression and nitrogenase activity could have an indirect effect on ammonium release. Similarly, it has been recently observed that more ammonium is excreted when a plasmid containing *nifA* under a constitutive promoter is introduced into the ammonium transporter AmtB mutant derived from the natural nitrogen-fixing bacterium *Pseudomonas stutzeri* A1501 [57].

Regarding the field of technology, in this work we showed that it is feasible to increase the ammonium available in the medium and hence the productivity of plants through the inoculation with recombinant bacteria expressing a heterologous nitrogenase (Figure 4, Figure 5, Figure 6, Figure 7, Figure 8, Table 1). We selected Pf-5 as the receptor of nitrogen-fixation genes because this bacterium is currently used as a commercial inoculant for biological control [88], [58]. Therefore, the recombinant strain Pf-5 X940 could increase the productivity of crops through different mechanisms. In addition, the technology of *nif* transfer used in this work can be applied to other host strains (Figure 4), thus opening the possibility to develop a new family of recombinant inoculants. On the other hand, we found that the inoculation with Pf-5 was effective both for dicots (alfalfa and Arabidopsis) and monocots (fescue and maize) (Figure 5, Figure 6, Figure 7, Figure 8). Therefore, we can speculate that the new family of recombinant inoculants could potentially be used in many commercial crops. In addition, we carried out several experiments that facilitate the release of the genetically modified organism Pf-5 X940: the clone Pf-5 X940 has only one insertion of cosmid X940 (Figure 2) and the genome walking describing the localization of this insertion (Figure S3).

Despite the great potential of this new technology, it is essential not to underestimate its complexities. In the technical area, all experiments were performed under non-agricultural conditions, so it cannot be ruled out that the recombinant strain may be displaced by indigenous microorganisms or not fix nitrogen in some soils with different physico-chemical features. In the regulatory context, the dysregulation of Pf-5 X940 may be too expensive because this strain has more than fifty transgens which can be allergenic and/or toxic to animals or humans. This problem could be reduced if the recombinant bacteria are not found in the aerial plant parts. In addition, before the release of Pf-5 X940 an evaluation of the ecological impact of its application on the microbial genetic structure is needed [25], [26], [30].

In conclusion, here we describe a new technology to develop nitrogen-fixing recombinant bacteria and its application to increase plant growth under nitrogen-deficient conditions, opening a new vista in agricultural biotechnology.

## Supporting Information

Figure S1 **Schematic representation of X940 cosmid containing** ***nif*** **genes from A1501.**
(TIF)

Figure S2 **The presence of** ***nifH*** **gene within the recombinant** ***Pseudomonas*** **strains.**
(TIF)

Figure S3 **Schematic representation of genome walking assay (X940 cosmid).**
(TIF)

Figure S4 **Effect of PST1307–1312 region in adaptation of** ***nif*** **genes to Pf-5.**
(TIF)

Figure S5 **Overlapping of** ***P. stutzeri*** **A1510 nitrogenase homology-modeled structure (light blue) onto** ***A. vinelandii*** **nitrogenase crystallographic structure (pdb-ID:1M1N) (green).** Fe(8)-S(7) cluster (left box), Fe(7)-Mo-S(9)-N cluster (right box), HCA (3- hydroxy-3-carboxy-adipic acid) and cysteines involved in the binding to the clusters are shown. This image was rendered with PyMOL (Python Molecular Graphics 1.3, 2009–2010, DeLano Scientific LLC, San Carlos, CA) also used to calculate the bond length between the clusters and cysteines sulfur (measured mean length for *A. vinelandii* = 2.3 Å and predicted mean length for *P. stutzeri* = 2.25±0.06 Å).
(TIF)

Table S1 **Primers used in this work.**
(DOC)

Table S2 **The statistical analysis of bacterial growth.**
(XLSX)

Dataset S1 **Nucleotide sequence of cosmid X940.**
(TXT)
