# Correction: Engineering *Pseudomonas protegens* Pf-5 for Nitrogen Fixation and its Application to Improve Plant Growth under Nitrogen-Deficient Conditions

**DOI:** 10.1371/annotation/279fe0d7-d9b1-4d05-a45a-5ff00b4606b7

**Published:** 2013-10-30

**Authors:** Lorena Setten, Gabriela Soto, Matteo Mozzicafreddo, Ana Romina Fox, Christian Lisi, Massimiliano Cuccioloni, Mauro Angeletti, Elba Pagano, Antonio Díaz-Paleo, Nicolás Daniel Ayub

In Table 1, the data in the bottom row was published with errors. Please see the corrected Table 1 here: http://www.plosone.org/corrections/pone.0063666.t001.cn.docx

