# Expression of concern: Engineering *Pseudomonas protegens* Pf-5 for nitrogen fixation and its application to improve plant growth under nitrogen-deficient conditions

**DOI:** 10.1371/journal.pone.0351791

**Published:** 2026-06-17

**Authors:** The PLOS One Editors

Concerns were raised following publication of this article [1,2] regarding the availability of the genetically modified bacterial strains developed in this study.

During editorial follow-up, authors MM, MC, and MA responded that, to the best of their knowledge, the bacterial strains are no longer available.

The *PLOS One* Editors are also aware that the following patent applications are indexed online with a filing date of November 22, 2012:

- 9657298: Recombinant nitrogen-fixing bacterial strain, inoculum containing the same and application methods
- CN104136599A: Recombinant nitrogen-fixing bacterial strain, inoculum containing the same and application methods

The Competing Interests statement for the article declares that no competing interests exist. MM, MC, and MA stated that at the time of submission and publication of [1], they were not aware of any patent, patent application, or procedure related to the intellectual property protection of the bacterial strains described in [1]. The *PLOS One* Editors have been unable to contact the remainder of the authors of [1] to clarify the existence of patents, products, or products under development related to the content of [1].

In light of the concerns above regarding the availability of the strains required to reproduce the reported results, and the patent applications that were not declared, the *PLOS One* Editors issue this Expression of Concern.
