# Supplementary figures and images for: Engineering Pseudomonas protegens Pf-5 for Nitrogen Fixation and its Application to Improve Plant Growth under Nitrogen-Deficient Conditions
Source: PLoS One. 2013 May 13;8(5):e63666. doi: 10.1371/journal.pone.0063666 (PMC3652814; doi:10.1371/journal.pone.0063666)

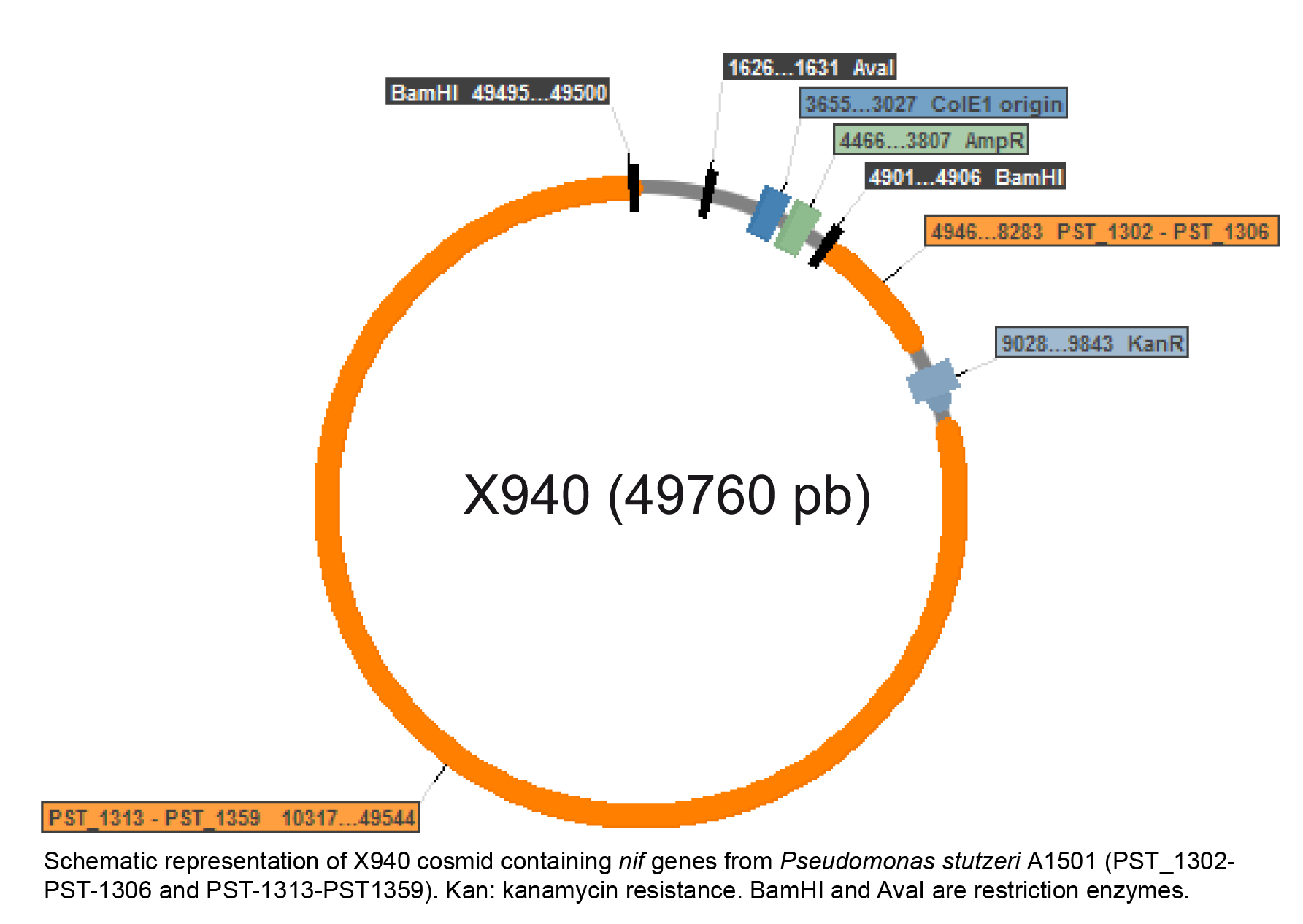

Supplement: Figure S1 — Schematic representation of X940 cosmid containing nif genes from A1501. (TIF) [file pone.0063666.s001.tif]

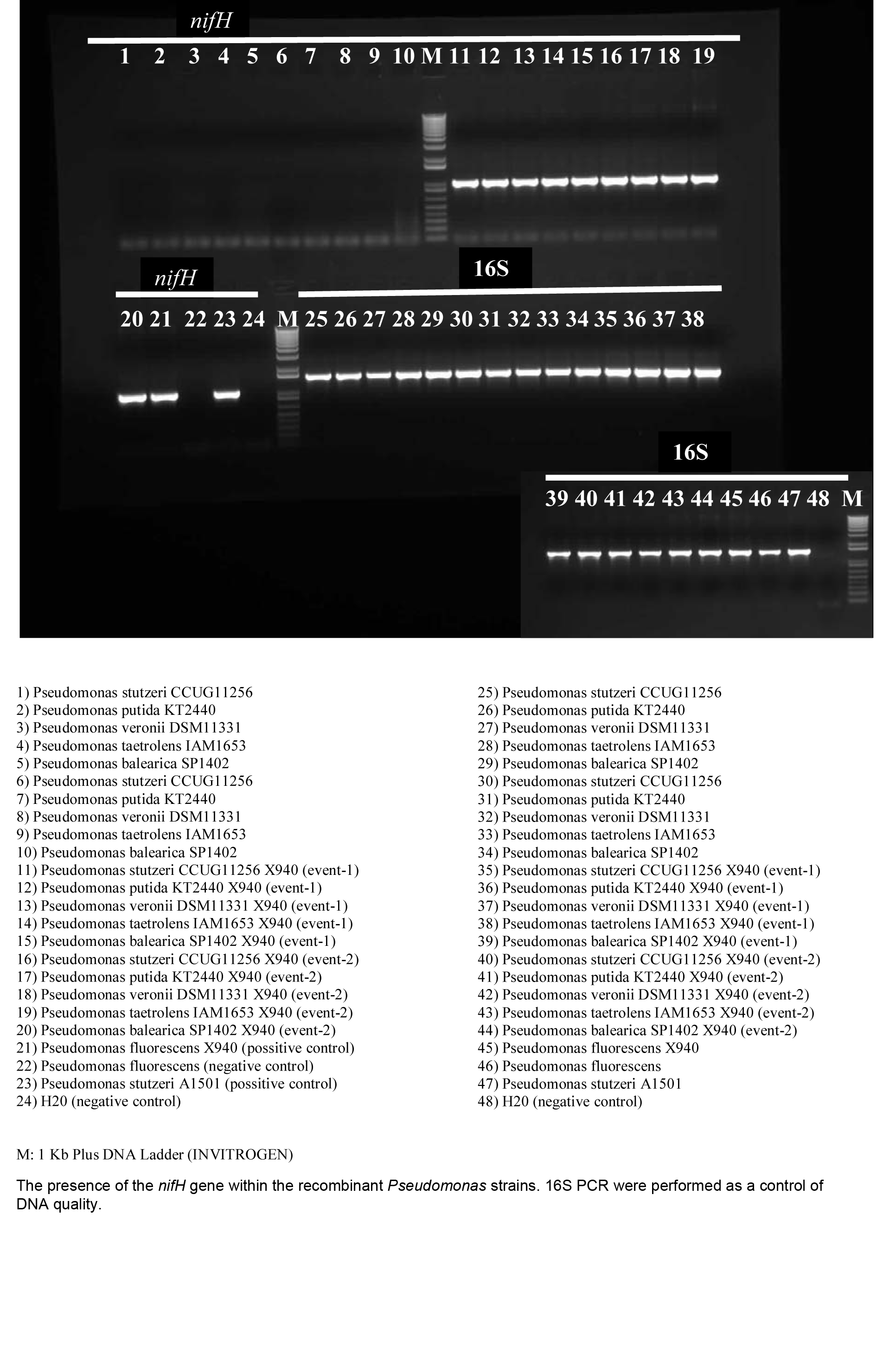

Supplement: Figure S2 — The presence of nifH gene within the recombinant Pseudomonas strains. (TIF) [file pone.0063666.s002.tif]

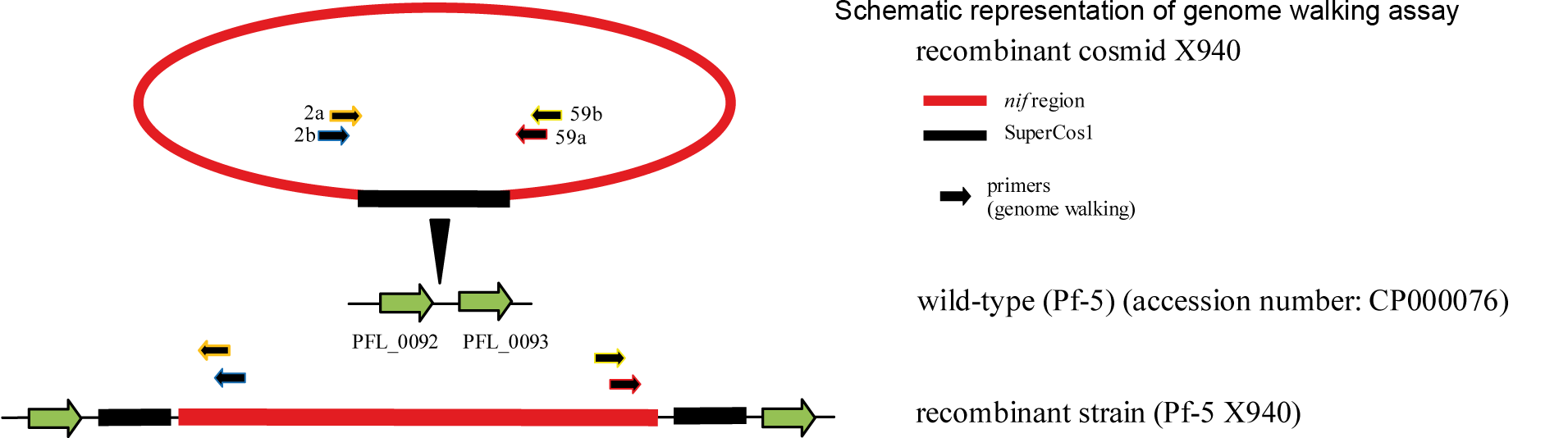

Supplement: Figure S3 — Schematic representation of genome walking assay (X940 cosmid). (TIF) [file pone.0063666.s003.tif]

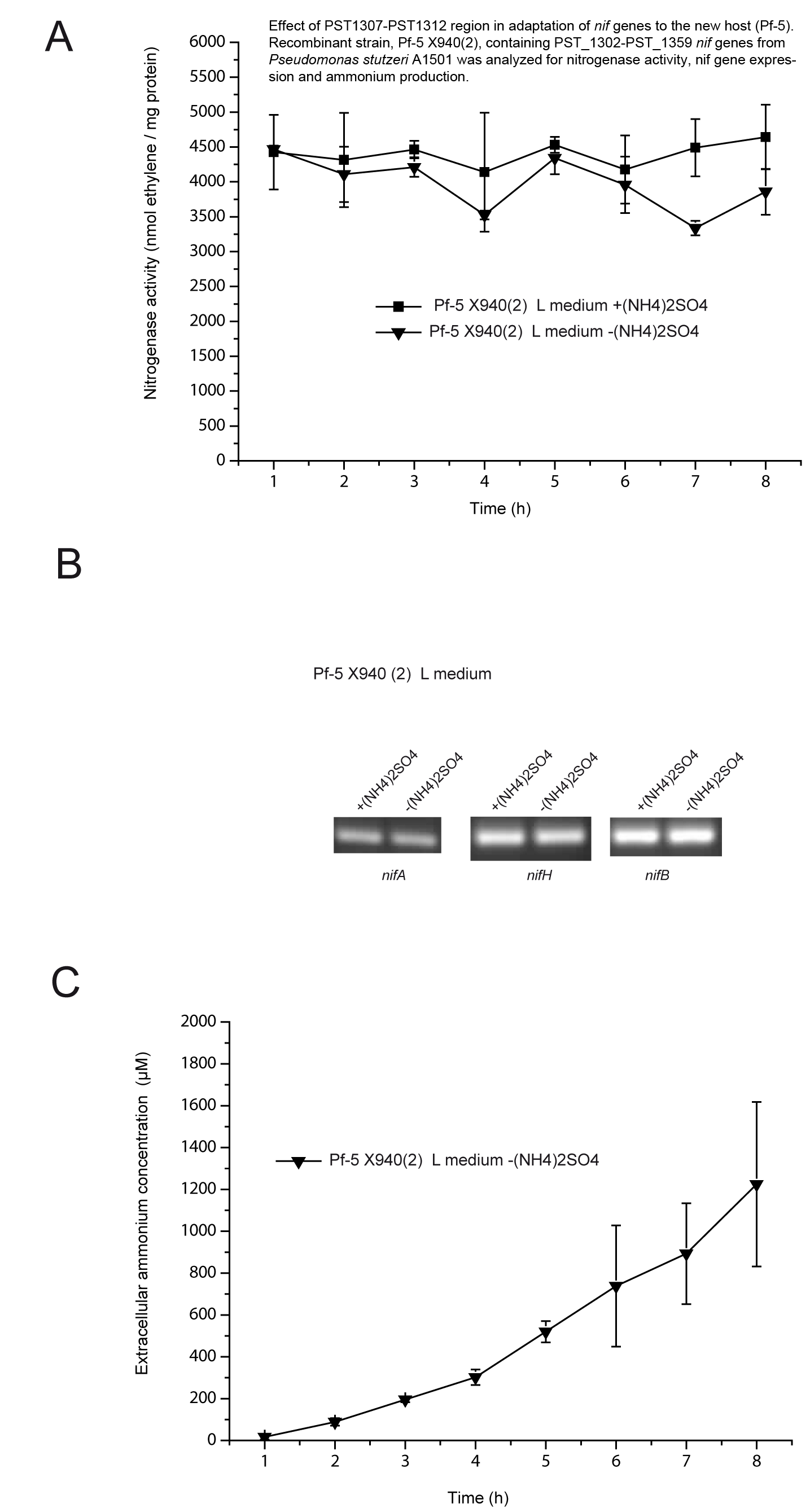

Supplement: Figure S4 — Effect of PST1307–1312 region in adaptation of nif genes to Pf-5. (TIF) [file pone.0063666.s004.tif]

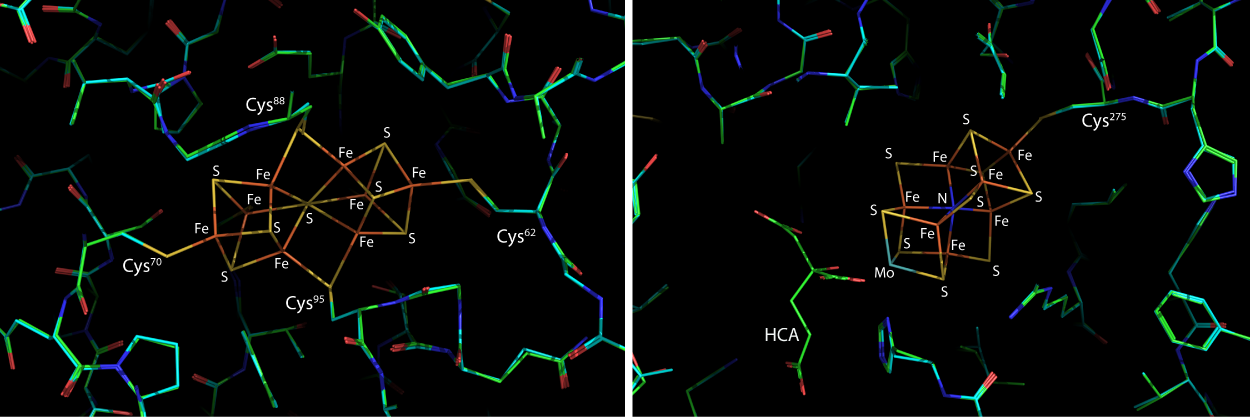

Supplement: Figure S5 — Overlapping of P. stutzeri A1510 nitrogenase homology-modeled structure (light blue) onto A. vinelandii nitrogenase crystallographic structure (pdb-ID:1M1N) (green). Fe(8)-S(7) cluster (left box), Fe(7)-Mo-S(9)-N cluster (right box), HCA (3- hydroxy-3-carboxy-adipic acid) and cysteines involved in the binding to the clusters are shown. This image was rendered with PyMOL (Python Molecular Graphics 1.3, 2009–2010, DeLano Scientific LLC, San Carlos, CA) also used to calculate the bond length between the clusters and cysteines sulfur (measured mean length for A. vinelandii = 2.3 Å and predicted mean length for P. stutzeri = 2.25±0.06 Å). (TIF) [file pone.0063666.s005.tif]
