# Supplementary material for: Engineering Pseudomonas protegens Pf-5 for Nitrogen Fixation and its Application to Improve Plant Growth under Nitrogen-Deficient Conditions
Source: PLoS One. 2013 May 13;8(5):e63666. doi: 10.1371/journal.pone.0063666 (PMC3652814; doi:10.1371/journal.pone.0063666)
Supplement: Table S1 — Primers used in this work. (DOC) [file pone.0063666.s006.doc]

**Table S1.** Primers

Name Sequece (5´XXX 3´)

A1 CGGGATCCCCGAATAGAGGTCTGTCCCCG BamHI restriction site is underlined

A2 CGGGATCCCCGGGGCGCTGGTGC BamHI restriction site is underlined

A3 CGGTCGACTCGGTGCGGCGCTCG SalI restriction site is underlined

A4 CGGTCGACGCCAAGGCCGCCCGC SalI restriction site is underlined

nifA1up AGTAGGCGGGCGACGATTTCCATC

nifA1low GCTGTACGACATGGACCTGCCCTTC

nifH1up GGAATCGGCAAATCCACCACGAC

nifH1low GGCCATTTCCATGATGGTGTTCTGC

nifBup ATCCCCTGCCTGGGGCTGAAC

nifBlow GCACCAGCCGCATACGTTCTCCTC

Gap-1up GGCAAAGGCAAGCTGAAGAACATCG

Gap-1low ACCGTTGATGATGCCGAACTTGTCA

p07 GAAGTAGTGGTGCGCCTCCTCGGAG

p13 CGGGTCGGGCGGCGCAGCGT

2a CTGGTGGCGCGACTCGATGCCG

2b TCGATGCCGCACTTGCGGCGGAA

59a GGCGCCTGGTTGCGACAGATCGCT

59b CACCGAGGAGCGGCTGGGCG

PST1302up CAGGCCGAGGATGATGTC

PST1306low GCTGGAGGTGTACCAGGAAA

N1 TTTAGAAACGCTGGCACAAA

N2 CGACGATGGTCATGTCTTCC
